# Determining genotype and antimicrobial resistance of *Salmonella* Typhi in environmental samples by amplicon sequencing

**DOI:** 10.1371/journal.pntd.0013211

**Published:** 2025-07-08

**Authors:** Catherine Troman, Samuel T. Horsfield, Dilip Abraham, Venkata Raghava Mohan, Sidhartha Giri, Satheesh Nair, Alexander G. Shaw, Zoe Dyson, Kathryn E. Holt, Nicholas C. Grassly

**Affiliations:** 1 Department of Infectious Disease Epidemiology & MRC Centre for Global Infectious Disease Analysis, School of Public Health, Imperial College London, London, United Kingdom; 2 European Molecular Biology Laboratory, European Bioinformatics Institute, Hinxton, United Kingdom; 3 Christian Medical College, Vellore, India; 4 Gastrointestinal Bacteria Reference Unit, UK Health Security Agency, London, United Kingdom; 5 Department of Infection Biology, Faculty of Infectious and Tropical Diseases, London School of Hygiene and Tropical Medicine, London, United Kingdom; 6 Wellcome Sanger Institute, Wellcome Genome Campus, Hinxton, United Kingdom; Advanced Centre for Chronic and Rare Diseases, INDIA

## Abstract

**Background:**

Estimates of the burden of typhoid fever due to *Salmonella enterica* serovar Typhi (*S.* Typhi) rely on data from clinical surveillance, which is rarely done in low income settings and is also limited by the poor sensitivity of the assays used and the reliance on health seeking by patients. Environmental surveillance for *S.* Typhi shed by symptomatic and asymptomatic individuals in wastewater offers a sensitive surveillance tool that could help to inform burden estimates. Sequencing *S*. Typhi direct from wastewater concentrates has the potential to identify circulating genotypes and associated antimicrobial resistance (AMR) genes, supporting public health interventions such as vaccination and antimicrobial usage.

**Methodology and principal findings:**

We designed a multiplex targeted amplicon sequencing protocol for genotyping and determining AMR in *S.* Typhi from wastewater samples, targeting SNPs that identify genotypes of interest and both chromosomal and plasmid-borne AMR. PCR products were sequenced using the Oxford Nanopore Technologies (ONT) MinION, and genotypes and AMR identified using the GenoTyphi program.

We tested this approach on samples from south India from both hospital outflow and wastewater collected from the community. All samples tested were suspected to be positive for *S.* Typhi following quantitative PCR for *ttr, tviB,* and *staG* gene targets. Out of 110 samples tested we were able to determine a genotype and/or AMR for 8. All samples that gave a genotype call suggested a genotype consistent with those found in clinical cases in India during the same time period and produced consensus sequences that clustered with *S.* Typhi when included in a phylogenetic tree.

**Conclusions:**

In this study, we provide proof of concept data for amplicon sequencing of *S*. Typhi in wastewater which with further optimisation could be used to complement clinical surveillance data or provide data on *S.* Typhi presence in the absence of clinical surveillance. This information can inform public health interventions, and the concept could be applied to other pathogens of interest for genotyping from environmental surveillance samples.

## Author summary

This study investigated the application of wastewater surveillance for detection of *S.* Typhi. Wastewater surveillance is of increasing interest for public health as a potential lower-cost alternative or complement to clinical surveillance. As well as confirming if *S.* Typhi is present in a sample, obtaining sequence information would allow the genotype and antimicrobial resistance present to be determined. Culturing *S.* Typhi from wastewater samples is difficult, preventing the application of traditional sequencing approaches using culture isolates. We therefore developed an amplicon sequencing protocol with a primer scheme targeting known genotype-defining single nucleotide polymorphisms and AMR genes that we tested on samples in south India.

This was implemented as a targeted multiplex PCR using four primer panels, with sequencing of the products performed on the Oxford Nanopore Technologies (ONT) MinION. We were able to identify the genotype and AMR profile for 8 of 110 samples previously shown by quantitative PCR to contain *S.* Typhi gene targets (*ttr, staG,* and *tviB*).

The results from this study show promise for the application of targeted next-generation sequencing to characterise *S.* Typhi in wastewater.

## Introduction

*Salmonella enterica* serovar Typhi (*S.* Typhi) is the primary causative agent of typhoid fever, with an estimated global burden of 9 million cases and 110 thousand deaths per year [1,2]. The current gold standard method for surveillance of typhoid fever relies on blood culture of symptomatic patients attending specialised hospitals or clinics which has a sensitivity ranging from 40-80% [3]. There are a variety of serological tests available based on the presence of *S.* Typhi-specific antigens, however these methods also vary in sensitivity and specificity [4]. Alternatively, molecular detection of *S.* Typhi by both PCR and q-PCR has been implemented for blood samples, targeting genes which can differentiate between *Salmonella enterica* serovars. However, these methods also tend to have poor sensitivity and may be difficult to employ for diagnosis in low-resource settings [5–8].

Irrespective of the method for diagnosing typhoid fever, surveillance of only symptomatic patients who attend a hospital or clinic can lead to underestimates of the burden in a community [9]. Environmental Surveillance (ES) based on testing wastewater and sewage samples can be used to assess the extent of *S.* Typhi circulation in a community, independent of healthcare-seeking behaviour or the development of symptoms, with successful use of environmental surveillance being seen in the cases of poliovirus and COVID-19 [10,11]. Data gathered could be used to complement clinical surveillance, provide data in the absence of clinical surveillance, contribute to disease burden estimates, or be used to evaluate the success of public health interventions such as vaccination or improvements to wash, sanitation and hygiene [12–14].

Studies have detected *S.* Typhi in water and wastewater samples using a variety of methods, such as culture, direct PCR or qPCR, or enrichment in specific media followed by PCR [13,15–17]. Using culture-based methods for ES can be difficult as isolation of *S.* Typhi can be variable and not as sensitive as when used for clinical samples [12,15]. Additionally, direct molecular detection by qPCR can be difficult due to gene targets being present in other organisms within the sample, thus reducing the specificity and reliability of the assay. For example, the *staG* gene used to detect *S.* Typhi in some studies is also found in some non-typhoidal *Salmonella* [5].

In addition to detecting *S.* Typhi, one consideration for public health intervention is the presence of antimicrobial resistance (AMR). AMR can be gained by mutation within the bacterial chromosome, with reduced susceptibility or resistance to fluoroquinolones and azithromycin, for example, being conferred by mutations in the *gyrA* and *acrB* genes respectively [18,19]. Antimicrobial resistance can also be acquired from a plasmid, with multidrug resistance in *S.* Typhi coming from plasmids such IncHI1 and IncY [20–22] with the IncHI1 plasmid sequence type 6 (PST6) being globally dominant in multiple drug resistant typhoid [23].

Sequencing *S.* Typhi in wastewater could address the challenge of poor specificity of qPCR by providing a sequence that can be checked against off-target organisms and also allow identification of the specific genotype present and its AMR gene profile by targeting amplification of the SNPs that define them. Sequencing of *S.* Typhi genomes has allowed the differentiation of different lineages and haplotypes using SNPs within certain genes [24,25]. In Wong *et al* (2016) a genotyping framework for defining *S*. Typhi lineages was presented using the combination of SNPs present throughout the core genome to separate *S.* Typhi into 4 primary clades, 16 clades and 49 sub-clades (genotypes) following a naming system with hierarchical numbering [26]. Additional SNPs and genotypes have been added over time with an update of the framework listing 87 clades and subclades [27]. The scheme also includes SNPs in the chromosome that are indicative of reduced susceptibility or resistance to antimicrobials and some SNPs to define sub-clades strongly associated with drug resistance. For example, 4.3.1.1.P1 is identified by a SNP unique to that cluster which typically has resistance to chloramphenicol, ampicillin, co-trimoxazole, fluroquinolones, and third-generation cephalosporins from both chromosomal mutations and a plasmid [28].

In this study, we have designed a multiplex amplicon-based sequencing approach for genotyping *S.* Typhi from wastewater samples. Targeted amplicon sequencing is preferred compared with metagenomic approaches because of the probable low abundance of *S.* Typhi in the sample and the high levels of competition from other DNA targets in wastewater. In our approach, multiplex PCR is carried out on DNA extracted from the ES samples, products are sequenced using Oxford Nanopore Technologies (ONT) nanopore sequencing, and we use the GenoTyphi scheme to allocate selected genotypes and AMR SNPs. We provide proof of principle data for the use of amplicon sequencing to identify genotype and AMR from environmental samples using *S*. Typhi DNA and apply the method to hospital and community wastewater samples collected in south India.

## Method

### Ethics statement

Samples from UKHSA did not require ethical approval. Ethical approval for the detection of gastrointestinal bacterial pathogens from specimens, or the identification, characterization and typing of cultures of gastrointestinal pathogens, submitted to GBRU is not required as it is covered by UKHSA’s surveillance mandate.

Samples from Vellore, India were covered by two ethics applications approved by the Institutional Review Board of Christian Medical College, Vellore 11170 [OBSERVE] dated February 28, 2018, and IRB Min No.11170. amended on 22 July 2020, IRB number A23, 22.07.2020.

### Samples

DNA from three cultured strains of *S.* Typhi was provided by colleagues at the UK Health Security Agency (UKHSA) for initial testing of the primer scheme at Imperial College London. One was a CT18 reference strain (3.2.1 genotype), as well as 388722 (H58-A, genotype 4.3.1 with a gyrA-S83Y mutation) and 388723 (H58-B, genotype 4.3.1 with no AMR mutations) both isolated by blood culture in 2014 from samples from Nepal and Malawi respectively.

Samples from two ES studies were tested in Vellore, India, to test the PCR and amplicon sequencing method. 16 samples from an *S.* Typhi ES pilot carried out between April 2018 and October 2019 in Vellore [29] were used to test the PCR and amplicon sequencing method (Table 1). These consisted of 8 community and 8 hospital samples, which we subsequently refer to as the “pilot” samples. 6.5L of wastewater was taken for the community samples and processed using a bag mediated filtration system (BMFS) [30], pellets from the concentration process described in [29] were stored at -70°C until extraction. For the hospital samples, 2.5L of wastewater from a hospital outflow was filtered through a 0.45µm membrane. The membrane was incubated in 10mL of selenite-F at 37°C for 24hr, 2mL of which was kept for long-term storage at -70°C with 1mL used as input for extraction. All samples selected for this study were originally suspected *S.* Typhi positive by singleplex qPCR targeting the *staG* gene with a Ct cut-off value of 35 [6,29]. A different volume and method were used for the hospital samples due to the long process of filtering *in situ* by BMFS not being feasible in the hospital locations. Stored samples were re-extracted using the Qiagen PowerfecalPro DNA kit (Qiagen) following the manufacturer’s protocol and re-tested by triplex qPCR [17](Table 1). Samples that came up negative after re-extraction were still used as they would be useful for testing negative or lower quality samples. Variation between the original sample result and Ct value for the *sta*G and the new triplex Ct value may be due to sample quality after being stored and re-extracted, or difference in sensitivity of the assay. There may also be a sampling effect due to the pellet used in the initial study being different to the pellet used in this study therefore causing variation in the observed Ct values.

**Table 1:** Samples selected from the pilot study for testing the amplicon sequencing approach. The singleplex Ct value is the value from when the samples were first tested targeting staG, the triplex Ct values are from the re-extracted sample following the protocol described in [17]. The Nanodrop values for sample concentration was measured for the re-extracted DNA. Where it says NA in the Ct column, the qPCR was not performed, where it has a dash, the target was past the limit of detection.

Sample	Ct (singleplex)	Ct (triplex)			Nanodrop ng/µl
		*staG*	*ttr*	*tviB*	
Community-1	25.85	–	–	–	16.7
Community-2	28.83	–	35.13	–	15.9
Community-3	29.12	35.62	32.42	34.12	15.9
Community-4	32.15	29.59	26.2	35.67	168.7
Community-5	33.32	35.97	28.15	35.89	25.5
Community-6	34.83	37.5	30.53	33.65	422.2
Community-7	–	NA	NA	NA	148.1
Community-8	–	NA	NA	NA	578.3
Hospital-1	23.8	–	–	–	37.5
Hospital-2	24.99	26.54	18.89	35.09	33.4
Hospital-3	25.22	31.8	22.83	30.3	22.2
Hospital-4	26.94	26.39	22.17	25.01	13.7
Hospital-5	29.08	–	19.34	–	9.8
Hospital-6	31.64	31.54	21.86	29.53	31
Hospital-7	32.46	36.74	22.52	34.14	18.7
Hospital-8	34.19	34.49	29.89	34.44	50.8

After testing the pilot samples, an additional 94 community wastewater samples from Vellore collected during 2021–2022 in an *S*. Typhi ES study [31,32] were also tested. We refer to these samples as the “ES study” samples. Two methods were used for sampling: grab sampling 1L of sewage followed by membrane filtration on a 0.45μm filter; and Moore swabs left *in situ* for 24–48 hours and incubated for 24hr in universal pre-enrichment broth (Neogen) which was then filtered on a 0.45μm membrane filter. DNA was extracted using the Qiagen PowerfecalPro DNA extraction kit (Qiagen) following the manufacturer’s protocol and DNA was eluted into 50μl of the elution buffer provided in the kit. Samples selected for sequencing were suspected positive for *S*. Typhi after triplex qPCR targeting *tviB, staG,* and *ttr* genes [17]. If all three targets were positive (giving a Ct value below 38 for *ttr* and 39 for both *staG* and *tviB*), the sample was considered positive for *S.* Typhi and put forward for sequencing.

### SNP site selection

A selection of SNPs from the genotyping framework in Wong et al (2016) were chosen for targeting in the PCR panel [26]. These included SNPs defining the primary clades 1, 2, 3, and 4, other higher-level clades, and a small selection of subclades of interest, for example the H58 clade (sub-clade 4.3.1 and its lineages 4.3.1.1, 4.3.1.2, and 4.3.1.3). The AMR SNPs lie within the *acrB*, *gyrA* and *parC* genes, which determine reduced susceptibility and resistance to azithromycin and fluoroquinolones respectively. Selected SNPs and their locations relative to the CT18 reference genome were obtained from GenoTyphi [26] and are listed alongside the primer sequences in Table 2. The incHI1 PST6 plasmid was also included in the selection of targets [21].

**Table 2:** Target SNPs, rationale for their selection, and their location in the CT18 genome, primer sequences, length, and melting temperature (Tm; calculated by RUCS) for all targets in the primer scheme, and the length of each resulting amplicon.

Primer Pool	SNP Target	Reason for SNP selection	SNP position(s) on CT18 genome	Forward primer sequence (5’-3’)	Primer length (bp)	Primer Tm (°C)	Reverse primer sequence (5’-3’)	Primer length (bp)	Primer Tm (°C)	Amplicon Length (bp)
A	0.0.1	High level clade	773487	TTTTCCATCTTAATTCATGCCTGGAT	26	61	CACTTTATTACTGCTCGGTTCGT	23	62	4224
	0.0.2	High level clade	1804415	CGTTGTCTGTGACACCATTCC	21	63	CCGGGTGCGTTACTATCCTATATTA	25	62	4978
	0.0.3	High level clade	1840727	ACTAGCGCTTTGATGGCCTTA	21	63	GTTAGCCGACTCTCGTATCCAA	22	63	5326
	0.1	High level clade	655112	CGTTATGTATCGCCCTTCTAAATTCAT	27	61	ATGCGCCCATGACTGAAACA	20	65	4590
	1	High level clade	316489	CCGTCACTAATCTGGATTTTGATCA	25	61	ACCAAATTTGCTTACAGGAAAAATAACA	28	60	4843
B	2	High level clade	2737027	GAAATTAGCGGCCTGGAAAAC	21	61	CGCCGTACTGAACAGGATATC	21	62	4760
	3	High level clade	3062270	TATTTAACGAAGATGGCCAATTGCT	25	61	GACGTTGAGGATTCCGGTGAT	21	64	4521
	3.1.1	Prevalent in West Africa	2732615	AAACTTTACATAGTGCTAAATCGTCGATT	29	61	ATTTCCCGGCTAGGCTCACT	20	66	4585
	3.3.2	Prevalent in Bangladesh	3498544	GAAGAAGCGCTTCGGACGAT	20	65	AGGACTGGAATTCCTGTATGTCTATATG	28	62	5042
C	4	High level clade	1615350	GTCGCCAGATCTTTTAATGACTGTT	25	62	CCCCTGTGTACGGAAGAACAC	21	65	5147
	4.3.1	Globally dominant clade (H58)	2348902	TGATATTCCCTTTCGTCGAACCTT	24	63	AGCTGCCATTTTTCTTCGTTATCAA	25	62	4695
	4.3.1.1	H58 lineage 1	1193220	CTGATGAAACCAACGGCAGATAAG	24	63	TTAAGCACTTTCTCCTGCACTGA	23	63	4720
	4.3.1.2	H58 lineage 2	3694947	GGGCTGAGTTGCTGTTTCTG	20	64	GTCTGGATTTTTGACGGATCAACAT	25	62	4555
	4.3.1.3	H58 lineage 3	561056	ATGGCGCGTAGACCAAACTG	20	65	ACCAGCGCGAAATTATTCACATC	23	63	4928
D	gyrA	AMR mutations	2333750, 2333751, 2333762	CCGCAACGGCAAAATAAATATCC	23	62	CCGAGCCATCCATTAAATTCTGAT	24	62	4267
	parC	AMR mutations	3196458, 3196459, 3196469, 3196470	ACTAATCGGAAAGAAAAGCGTTTCA	25	61	GGCGACGTACTGGGTAAGTATC	22	64	4768
	PST6	Plasmid know to confer AMR	N/A	TGACCCCTACATTACAGGTAGTCT	24	63	CCGAAAAATGTGACGGTGTCA	21	63	4628
	acrB	AMR mutations	523109	TCTGACAGGAGAAAATAGCTAGGA	24	61	CGAAGGTCGATCTGGTGACAAG	22	65	3854
	4.3.1.1.P1	XDR sub-lineage of H58 lineage 1	955875	TGTTGAACCGTCAAAATTTACGTTT	25	60	GTCTTTACCCAAGCCCAGAATATAG	25	62	4730

### Primer design

Primers targeting regions around selected SNPs were designed using RUCS [33]. A selection of *S.* Typhi genomes or plasmids were provided as a positive reference set against which primers were designed. The positive set also included genomes for all lineages described in the GenoTyphi scheme [26]. Additionally, a negative reference set was provided to remove primers with non-specific binding, including genomes from non-typhoidal *Salmonellae* and non-*Salmonella* species. Accessions for all positive and negative genomes and plasmids are given in S1 Data. To ensure the primers only spanned the region of interest, a 10kb region of the CT18 genome surrounding each target SNP was included in the positive set and the remainder of the genome was put in the negative set.

RUCS was run with 20 bp minimum, 25 bp optimum, and 30 bp maximum primer length with 2 maximum 3’GC bases, and 57ᵒC minimum, 60ᵒC optimum, and 62ᵒC maximum primer melting temperature. The number of primer pairs to return was set to 10,000 and amplicon size range set at 300–10,000 bp. Candidate primers were filtered based on their specificity using BLAST [34]. Pairs that aligned to the most *S.* Typhi genomes and the fewest off-target genomes were taken forward, then the primer pairs were selected to give the most consistent amplicon size. The final candidates were checked again for specificity using the primer-BLAST tool [35]. The final primer sequences and other primer details are listed in Table 2.

### PCR

Primers were initially tested in singleplex and multiplex reactions (as a single panel or as 2 or 4 panels), and the most highly multiplexed PCR that gave the most even coverage of amplicons was taken forward for further testing.

PCR reactions were set up in a final volume of 25μl using LongAmp Taq 2x Master mix (NEB), bovine serum albumin at a final concentration of 0.4µg/µl, and a final concentration of 0.4μM per primer. 5μl of sample DNA was used as input for each reaction.

Targets were amplified using a touchdown PCR to improve primer binding specificity. After an initial denaturation at 94ᵒC for 2 minutes, there were 13 cycles of: 94ᵒC for 30s, 63ᵒC for 1m (decreasing by 1ᵒC each cycle), and 65ᵒC for 5m, then 27 cycles of: 94ᵒC for 30s, 50ᵒC for 1m, and 65ᵒC for 5m, then finally 65ᵒC for 5 minutes. Different temperatures for the touchdown PCR were tested initially, all with a starting temperature of the average Tm as calculated by RUCS. Finishing the touchdown at 50°C gave the fewest products shorter than the target length. PCR products were purified using a 0.45 ratio of AMPureXP magnetic beads (Beckmann-Coulter) to remove any short off-target amplicons, so for a 25μl PCR reaction 11μl of AMPureXP was used.

### Sequencing

Amplicons were quantified using a Qubit fluorometer with the broad range dsDNA assay kit (Thermofisher). For each sample the four reactions were pooled equally to total approximately 200 fmol. Each sample was prepared for sequencing using the ONT native barcoding expansion (EXP-NBD104, EXP-NBD114, or EXP-NBD196) and ONT protocol for native barcoding amplicons. Each barcoded sample was quantified by Qubit and pooled to give a library totalling 1μg.

The pooled library was adapted for sequencing using the AMII in the native barcoding expansion and buffers in the sequencing by ligation kit (SQK-LSK109). Sequencing was carried out using an R9.4.1 flow cell on a MinION Mk1B connected to a laptop (Dell Precision 7540 running Ubuntu 20.04.6) with a GPU (NVIDIA Quadro RTX 3000) capable of high accuracy basecalling and demultiplexing in MinKNOW (version 21.06.13, guppy version 5.0.16). Adaptive sampling was also enabled during the run, providing the CT18 genome and PST6 plasmid sequences for enrichment of *S.* Typhi targets. If the required computing power for real-time analysis was not available, basecalling and demultiplexing were carried out post-sequencing using the command line version of guppy.

Further details on the runs including total output, run length, and N50 are given in Table A in S2 Data.

### Sequence analysis with RAMPART and GenoTyphi

Demultiplexed reads in fastq format were mapped to a set of reference genomes (Table B in S2 Data) including *S.* Typhi chromosomal sequences, PST6 plasmid sequences, non-typhoidal *Salmonella* species, and some non-*Salmonella* species using RAMPART (https://artic.network/rampart). The minimum read identity was set to 90% to prevent incorrect mapping but allow for some sequencing errors and mutations. Reads that mapped to *S.* Typhi were length filtered (3000–6000 bp) to include only the expected amplicon lengths and exported to fastq, giving a separate file per sample.

Exported fastq files were mapped to the CT18 reference genome (accession: AL513382.1) using minimap2 with the *-x map-ont* option to optimise mapping for nanopore reads. The resulting sam file was sorted and converted to a bam file using samtools v1.14 [36] which was then used as input for a version of GenoTyphi edited to only include SNP information for the SNPs targeted in this primer scheme (available at: https://github.com/typhoidgenomics/genotyphi/blob/main/mappingbased/genotyphi_ampliseq.py). The bam file and CT18 reference genome are used as input to GenoTyphi which creates variant call files (VCF) which are then compared to the list of known genotyping and AMR SNPs. GenoTyphi outputs a tab-delimited file with the following columns: Final call, a final call of a genotype to the highest level that could be determined (or a call of no SNPs found); Final Call support, a support value representing the proportion of reads containing SNPs to support the final call; Subclade to detail the lowest subclade with SNPs detected; Primary Clade to detail which primary clade was defined by the SNPs detected; Support Subclade and Support Primary Clade to give the support values for the previous two columns; Number of SNPs gives the total number of SNPs seen across the reads for the sample; AMR Mutations, details which AMR defining SNPs were detected.

For closer inspection of any off-target or unmapped reads, Geneious Prime 2022.2.1 (https://www.geneious.com) was used to map reads to the reference genome set.

### Consensus sequence generation

To confirm the validity of the genotype calls, consensus sequences were constructed using the amplicon sequences for each sample and phylogenetic trees were inferred to show how the consensus sequences relate to known *S.* Typhi and non-typhoidal *Salmonellae*. The purpose of the trees, rather than defining genotype was to check that the reads contributing to the genotype calls were actually from *S.* Typhi. For each sample giving a genotype call, consensus sequences for each amplicon target were built from the filtered reads output from RAMPART. The consensus was built using iterative mapping and polishing using racon v1.4.7 [37] and medaka v0.10.0 [38]. First the reads were mapped using minimap2 (-x map-ont) to a fasta file containing all reference amplicon sequences. The resulting paf file was passed through racon (-m 8 -x -6 -g -8 -w 500) which produced a fasta file for using as a reference for the next mapping iteration. This was done four times before running the final racon output through medaka consensus using default parameters to create a corrected consensus sequence for each target amplicon.

The consensus sequences for each target were aligned in Geneious using the MAFFT algorithm [39] with default settings and the resulting alignment used to create a neighbour-joining tree with the Geneious tree builder with default settings. As well as the sample consensus sequences the alignments and trees included known *S.* Typhi and non-typhoidal *Salmonella* serovars for comparison (Table C in S2 Data). 16 *S*. Typhi genomes from clinical isolates from India from 2018-2019 [40] were downloaded from Pathogenwatch (https://pathogen.watch/genomes). Final tree visualisation and editing was performed using the online tool Microreact [41].

## Results

### PCR optimisation

Different primer pooling approaches were tested, including combining all primers into a single pool, splitting into two pools, or splitting into four pools (S1 Fig).

**Fig 1:**
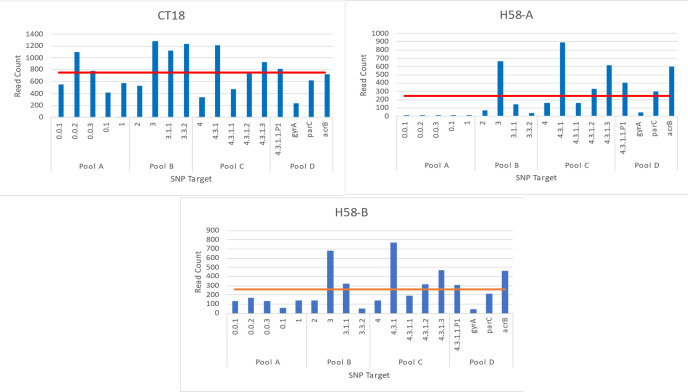
Distribution of reads across the SNP targets for the control DNA samples. Each bar is the total number of reads sequenced for each target amplicon, shown for CT18, H58-A, and H58-B. Targets are organised by primer pool. The red line indicates the average number reads for a target for each sample.

Overall, multiplex PCR with 4 primer pools gave the most even distribution of amplicons when tested on the control DNA and was used for further testing on the pilot and ES study sample DNA. When using the CT18 control DNA, 99% of all full-length reads mapped to *S*. Typhi, whilst this was 98% and 97% for H58-A and H28-B respectively. Although the 4-pool approach gave the best distribution, the distribution of sequencing reads across the targets still varied between the samples, especially the H58 strain A which had fewer than 15 reads for each target in Pool A, well below the average read count of 245 reads (Fig 1). Since the genotyping SNPs targeted in Pool A are less common, especially in the initial study site of Vellore, the lower coverage for those targets was accepted.

When run through GenoTyphi, all three samples gave the correct genotype call, with the H58 samples classified at a finer resolution (4.3.1.1) than previously reported (4.3.1) (Table 3). In all three control DNA samples, the primer set for the PST6 plasmid did not amplify, this is because the strains do not contain a PST6 plasmid.

**Table 3:** GenoTyphi output for all control samples and pilot samples. The plasmid results are not listed, however no samples were determined to have the PST6 plasmid present. Where no SNPs were detected, or no reads mapping to the target, GenoTyphi outputs “No SNPs encountered against expected reference. Wrong reference or no SNP calls?”.

Sample type	Final call	Final call support	Subclade	Primary Clade	Support Subclade	Support Primary Clade	Number of SNPs	Called AMR mutations
CT18 control	3	1		3			23	
H58-A control	4.3.1.1	0.95	4.3.1.1	4	0.98	0.97	23	gyrA-S83Y
H58-B control	4.3.1.1	0.96	4.3.1.1	4	0.99	0.98	23	
Community-1	3	1		3			1	
Community-2	No SNPs encountered against expected reference. Wrong reference or no SNP calls?							
Community-3	3	1		3			1	
Community-4	3	1		3			14	
Community-5	3	1		3			4	
Community-6	No SNPs encountered against expected reference. Wrong reference or no SNP calls?							
Community-7	3	1		3			9	
Community-8	No SNPs encountered against expected reference. Wrong reference or no SNP calls?							
Hospital-1	3	1		3			1	
Hospital-2	2	1		2			15	
Hospital-3	4.3.1.2	0.88	4.3.1.2	3	0.88		20	gyrA-S83Y
Hospital-4	4.3.1.1	0.48	4.3.1.1	4	0.91	0.52	22	gyrA-S83F
Hospital-5	3	1		3			11	
Hospital-6	4.3.1.1	0.12	4.3.1.1	4	0.47	0.25	19	gyrA-S83F
Hospital-7	4.3.1	0.96	4.3.1	2	0.96		20	gyrA-S83F
Hospital-8	4.3.1	0.72	4.3.1	4	1	0.72	14	

### Sequencing of the pilot samples and ES study samples

#### GenoTyphi result and consensus trees.

Overall, 6 of the total 110 (5.5%) samples tested in Vellore had a reliable genotype call from GenoTyphi. Of the 8 pilot community samples tested, none gave a genotype call past the primary clade, and three had no detectable SNPs (Table 3). Despite the Final Call support of 1 for all of the samples with a final call of 3, the Primary Clade support column is empty (Table 3) suggesting there were too few or no reads for the amplicon present. This unreliable call of genotype 3 occurred when the analysis software detected reads mapping to the reference genome but no SNPs to distinguish it from the reference, so it assigned the reference’s primary clade, 3.

Of the 8 pilot hospital samples tested, five gave a genotype with identification past the primary clade with support values (indicating the proportion of reads containing the SNP of interest) ranging from 0.12 to 0.96 (Table 3). These were all H58 types (4.3.1, 4.3.1.1, and 4.3.1.2), and four had SNPs in the *gyrA* gene indicative of fluoroquinolone non-susceptibility. Of those that had a final call of 4.3.1.1 or 4.3.1.2, the SNP for 4.3.1 was also present, strengthening confidence in the genotype call. However, multi-level lineage information is not included in the GenoTyphi output and does not contribute to the final support value which is the product of the subclade and primary clade support values (Table 3).

Of the 94 ES study samples, 79 (84%) had an unreliable final call, similar to those seen in the Community samples in Table 3. Just one sample (Study-43) had a final call of genotype 4.3.1.2 with a Final Call and Subclade support value of 1. Two samples also had SNPs for AMR genes, one with gyrA-S83F, and one parC-E84K. An overview of these GenoTyphi results can been seen in Table 4. The called genotypes in the 4.3.1 clade for both sets of samples are consistent with those found in clinical isolates in the same years [40,42].

**Table 4:** Summary of GenoTyphi output from Vellore ES Study samples.

Final Call and AMR detections	Number of Samples	Support for Primary clade or subclade
4.3.1.2	1	1
gyrA-S83F	1	NA
parC-E84K	1	NA
Unreliable final call	79	NA
No SNPs Encountered	14	NA

**Fig 2:**
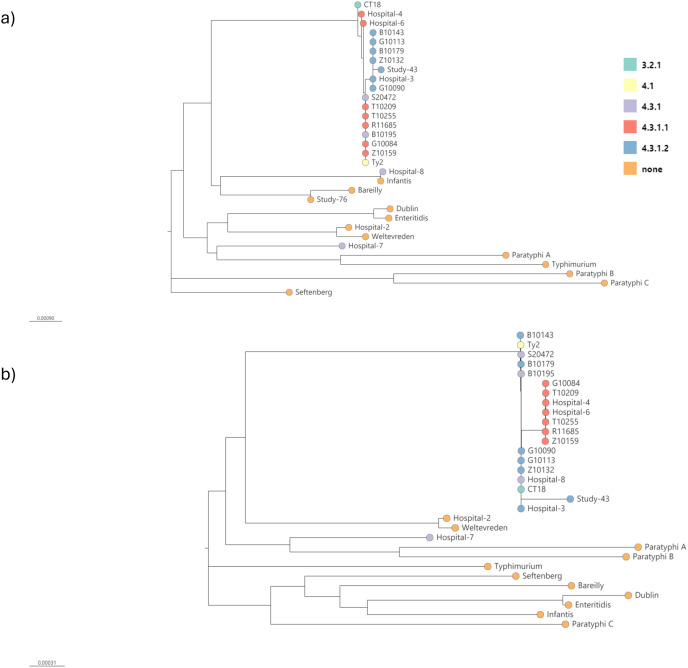
Neighbour joining trees for amplicon consensus sequences. Trees are shown for a) 4.3.1.2 amplicons b) 4.3.1.1 amplicons. Each sample and reference is marked by a coloured circle depending on its final call from GenoTyphi (or none for those with an unreliable call or that are not S. Typhi).

Fig 2 shows the phylogenetic trees built from the amplicon consensus sequences from all samples that produced consensus sequences for the 4.3.1.1, and 4.3.1.2 targets. The samples that were called as 4.3.1.2 (Study-43 and Hospital-3) or 4.3.1.1 (Hospital-4 and Hospital-6) had consensus sequences for those amplicons that clustered with *S.* Typhi isolates of the same lineage (Fig 2), increasing the reliability of the genotype calls. The samples that produced a consensus sequence for 4.3.1.1 or 4.3.1.2 but gave an unreliable genotype call (Study-76 and Hospital-2) did not cluster with the other *S*. Typhi sequences, supporting the decision that the call was unreliable and that the samples may not contain *S.* Typhi.

#### Organism mapping.

**Fig 3:**
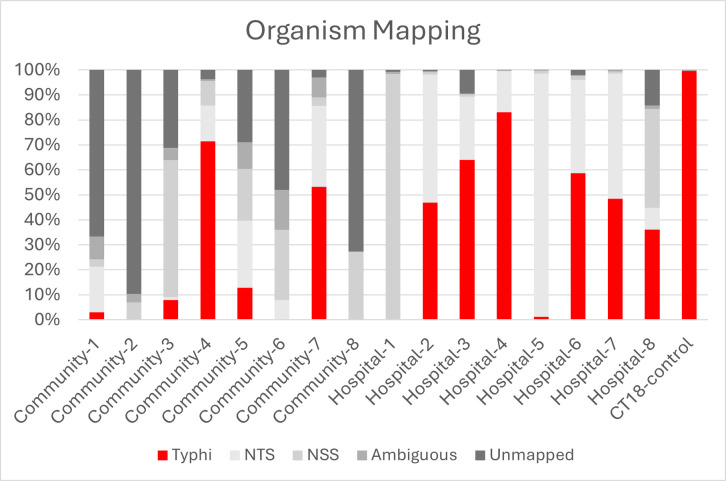
Mapping of pilot sample full-length reads to the reference set. The references include Salmonella Typhi (Typhi), non-typhoidal Salmonellae (NTS), and non-Salmonella sp. (NSS). Reads are shown as a percentage of the total for each sample.

The full-length (3000–6000 bp) reads for the pilot study samples were mapped to the RAMPART reference set in Geneious using minimap2. The majority of reads from the community samples were either unmapped or mapped to non-typhoidal Salmonellae and non-*Salmonella* species (Fig 3) with a median percentage of reads mapped to *S.* Typhi of 5.5% (IQR 43.1%). This non-specific amplification of non-*S.* Typhi sequences could explain the lack of detectable SNPs in these samples (Table 3). In contrast, the hospital samples had a larger proportion of reads mapping to *S.* Typhi genomes but there was still noticeable variation between samples (Fig 3) with the median mapped percentage of 47.7% with an IQR of 52.8%.

The ES study samples had similar compositions to the pilot community samples, with a median 4.6% of full-length reads mapping to *S.* Typhi (IQR 13.3%). Mapping the reads to the off-target reference set in Geneious showed that most off-target mapping was to non-*Salmonella* species, with the highest percentage mapping most often to *Enterobacter sp*. due to mis-priming of the 3.3.2 primer set.

For all runs in the ES study, the median sequence length was low (0.5-1.5kb), meaning that the majority of reads were much shorter than the target length. Among these reads, most that mapped to a reference produced an amplicon 1.8kb long mapping to *Klebsiella pneumoniae* (mis-priming of 3.1.1R and 2F). Many reads also mapped to *Aeromonas veronii* and *Citrobacter freundii* both of which could be attributed to mis-priming of the 3.3.2F and 2F primers forming an 850 bp product. Another mis-primed amplicon seen mapping to *Aeromonas veronii* was a 998 bp product produced by 4.3.1.2F and 4.3.1.1F.

All samples generated unmapped reads. Particularly high proportions were observed in the ES Study samples (median 68%, IQR 21%) and community pilot samples (median 76%, IQR 18%), whereas the hospital pilot samples had a lower proportion of unmapped reads (median 26%, IQR 33%). These unmapped reads included reads that did not pass the quality threshold for mapping or did not map to a genome in the reference set. Many unmapped reads were shorter than the target amplicons at <600 bp in length so did not pass the initial length filter in the analysis.

#### Read distribution.

**Fig 4:**
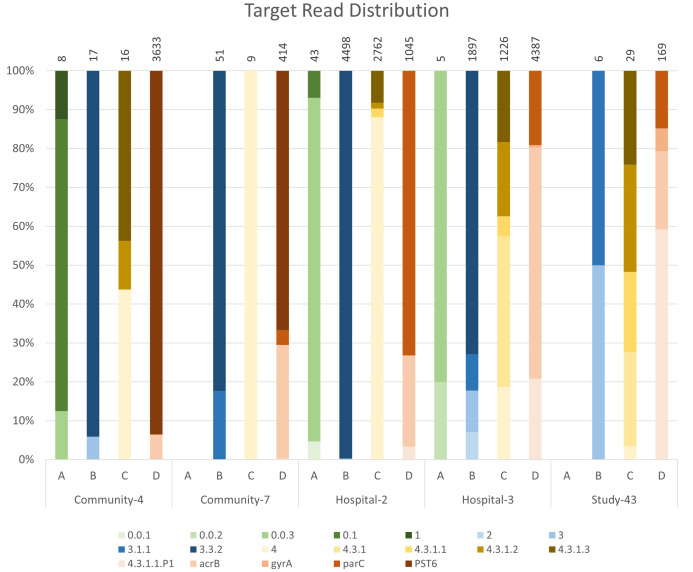
Distribution of full-length reads mapping to each target for a subset of pilot samples that gave a call in GenoTyphi and for the ES study sample that was called as 4.3.1.2. Each sample is divided into the four primer pools and the distribution is shown as a percentage of the total number of reads for each primer pool. Above each bar is the total number of reads for that primer pool.

For reads that did map to *S.* Typhi, the distribution of reads mapping to the individual primer targets varied across the samples (Fig 4). Fig 4 shows the distribution of the number of reads for each primer target for a subset of the pilot samples with >1 target SNP, and for the single ES study sample with genotype 4.3.1.2. The distribution for each pool varied between samples and not all targets had reads mapping in every sample. For all samples there were fewest reads (<1%) mapping to targets in Pool A, with some samples, such as Study-43, having no reads mapping for the whole primer pool (Fig 4).

Although very few reads in the ES study samples mapped to *S.* Typhi (median 4.6% full-length reads), of those that did, the majority were the amplicons targeting 3.3.2, *acrB*, and the PST6 plasmid. The 3.3.2 and *acrB* targets were also most common in the pilot samples.

## Discussion

Environmental sampling and detection of pathogens and AMR genes in wastewater complements clinical disease surveillance or providing data in the absence of clinical surveillance. In this study, we have demonstrated an amplicon sequencing approach to genotype *S.* Typhi in wastewater samples and detect AMR genes.

We adapted a whole genome SNP-based genotyping scheme [27,26] to enable differentiation of globally important *S.* Typhi lineages using amplicons targeting a subset of these SNPs (18 out of 96). Since not all known genotypes are targeted in the primer scheme, the absence of a genotype call does not mean that *S.* Typhi is not present. However, we were able to confirm *S.* Typhi in 6 of 110 (5.5%) environmental samples tested with confident genotype predictions (Tables 3 and 4) supported by a phylogenetic approach based on the amplicon consensus sequences (Fig 2). As well as genotype calls, 6 samples had SNPs in the chromosome indicative of antimicrobial resistance, meaning this method could be used to monitor the spread of AMR genes associated with *S.* Typhi lineages. Identifying genotypes and circulating AMR can inform epidemiological studies, enable modelling of multi-drug resistant lineage spread and public health risks, inform local, national and international usage of antimicrobials, and motivate the introduction and usage of typhoid vaccines in high burden areas.

The difference in results and amplification of hospital and community samples in the two sample sets could be reflective of the different sampling strategies. The pilot community samples were processed by BMFS whereas the hospital samples by membrane filtration, both of which also used different starting sample volumes (6.5L vs 2.5L). The more recent ES study samples again were processed differently by membrane filtration of grab samples or Moore swab eluates. The different sample volumes combined with the different sample processing strategies, could affect the chances of recovering target DNA from the samples due to the sampling effect of varying sample volumes and the level of concentration from the processing methods. This highlights the importance of optimised sample collection and processing strategies for the desired downstream application and the need for unified sampling strategies to allow comparison between sites.

Despite the equal concentration of each primer set in the PCR reactions and relatively even distribution when being tested on control DNA, the read distribution across the ES samples was highly variable (Fig 4). As well as variations in performance of the different primer pairs, this could be due to the amount of off-target amplification from some primer pairs, or combination of primers from different pairs, reducing on-target *S.* Typhi reads. It may also be due to the possible presence of inhibitors in the sample carried over from DNA extraction. Since the amount of interference from other organisms or inhibitors in the sample can vary, it is unlikely that altering the primer concentrations according to amplicon abundance in the samples in this study will improve the result. One way to improve sensitivity could be to target shorter amplicons to make the PCR more sensitive, or to reduce the number of primer pairs by including more than one SNP target per amplicon. The latter would require a more detailed SNP analysis since the SNPs used in the GenoTyphi scheme are mostly too far apart in the genome for >1 SNP to be targeted by a single amplicon. If it is possible to target multiple genotyping SNPs in a single amplicon, it would also be possible to target more known genotypes without having too many primer sets in a single multiplex.

Although the primers were originally designed to be specific to *S.* Typhi with a target minimum of 2 mismatches to related but off-target sequences, the high prevalence of off-target amplification, especially in the ES study samples, greatly reduced the number of on-target reads. In some cases, this is the result of primer binding despite more than two mismatches and so could be overcome with further optimisations of the PCR set up and cycling. Options include carrying out the reactions in single-plex to avoid primer interactions outside of the target primer pairs (although this would increase the hands-on time and cost of the method), enriching *S.* Typhi before PCR possibly by using a specific broth or using beads to capture *S.* Typhi, improving primer design to minimise the risk of unplanned primer combinations amplifying off-target genomes, and nanopore adaptive sampling to eject off-target amplicons during sequencing.

The large number of reads in some samples that did not map to *Salmonella* or other genomes included in the reference set could be due to two things. Firstly, the quality of the individual reads may be poor (errors in the read can affect whether it passes the threshold for mapping to a reference), which will be improved over time as ONT chemistry is updated and basecalling models improved. Since carrying out this work, ONT has released new kit14 chemistry which boasts a highly improved read accuracy in addition to the ability to duplex basecall, which predicts paired reads to increase read accuracy by creating a consensus of the two. Secondly, the breadth of the reference set will affect the number of mapped reads. As these are environmental samples there will be a very large diversity of organisms present, and the extent of off-target amplification can vary between samples (Fig 3). The reference set used in RAMPART currently focuses on *S.* Typhi genomes (as this is the target) and a small selection of other non-typhoidal *Salmonella* or non-*Salmonella* species. To reduce the number of unmapped reads, the reference set for RAMPART could be expanded to help understand what is causing the off-target amplification and inform primer optimisation. However, it is unlikely this would affect the number of reads mapping to *S.* Typhi.

We have demonstrated proof of principle for the use of amplicon sequencing from wastewater samples to confirm detection, and genotype and provide the AMR gene profile of S. Typhi. Having this information from environmental surveillance samples can help to confirm the presence of *S.* Typhi in the samples. Further optimisation of the primers and protocol are ongoing to improve sensitivity by identifying alternative, linked SNP targets to allow genotyping, and to reduce the size of the target amplicons. Following method improvement, validation and assessment of the limit of detection, this approach could be used in low-income settings to determine local *S.* Typhi genotypes and AMR gene profiles as an alternative or addition to clinical surveillance. Targeted next-generation sequencing using ONT platforms is attractive for this purpose because of the low start-up and follow-on costs. Nonetheless, substantial investment and training will be required if this method is to be used more widely. Integration with surveillance and targeted sequencing of other bacterial pathogens of interest may help to reduce costs and extend the utility of this approach. For example, detection and sequencing of *Salmonella* Paratyphi A that can also be delineated with a SNP-based approach, using the paratype scheme [43]. This integrated approach could be used to identify bacterial pathogens circulating or emerging in a community and their AMR gene profile even in the absence of clinical isolates, which may help inform usage of specific antimicrobials and guide vaccination where MDR or XDR strains are most prevalent.

## Supporting information

S1 Data Spreadsheet containing accessions numbers for all references used for running RUCS. Separate tabs are on the sreadsheet for the positive chromosomal references, positive plasmid references, and all negative references.
(XLSX)

S2 Data Table A. Sequencing run summary details for runs performed on the pilot study samples and the ES study samples. Table B. *Salmonella* Typhi, non-Typhoidal *Salmonellae*, and non-*Salmonella* organisms included in the reference set for mapping the sequencing reads. Table C. *Salmonella* Typhi and non-Typhoidal *Salmonellae* included in the consensus trees. All isolate sequences derived from Pathogen Watch originated in India and were dated between November 2018 and April 2020.
(DOCX)

S1 Fig Amplicon mapping for different primer pooling approaches. a) Single pool of all primers b) Primers split into two pools, Pool 1 and Pool 2 c) Primers split differently into two pools, Pool 3 and Pool 4.
(PDF)
