# Supplementary material for: Determining genotype and antimicrobial resistance of Salmonella Typhi in environmental samples by amplicon sequencing
Source: PLoS Negl Trop Dis. 2025 Jul 8;19(7):e0013211. doi: 10.1371/journal.pntd.0013211 (PMC12237024; doi:10.1371/journal.pntd.0013211)
Supplement: S2 Data — Table A. Sequencing run summary details for runs performed on the pilot study samples and the ES study samples. Table B. Salmonella Typhi, non-Typhoidal Salmonellae, and non-Salmonella organisms included in the reference set for mapping the sequencing reads. Table C. Salmonella Typhi and non-Typhoidal Salmonellae included in the consensus trees. All isolate sequences derived from Pathogen Watch originated in India and were dated between November 2018 and April 2020. (DOCX) [file pntd.0013211.s002.docx]

Table A. Sequencing run summary details for runs performed on the pilot study samples and the ES study samples.

| **Location** | **Run Number** | **No.Samples** | **Kit** | **Flow cell** | **Run length** | **MinKNOW version** | **Guppy version** | **Basecall model** | **N50 (kb)** | **Total reads** |
| --- | --- | --- | --- | --- | --- | --- | --- | --- | --- | --- |
| CMC, Vellore | Pilot-1 | 6 | SQK-LSK109, EXP-NBD196 | FLO-MIN106 | 2h | 21.06.13 | 5.0.16 | High Accuracy | 4.6 | 529.58k |
| CMC, Vellore | Pilot-2 | 11 | SQK-LSK109, EXP-NBD196 | FLO-MIN106 | 6h | 21.06.13 | 5.0.16 | High Accuracy | 3.89 | 1.64M |
| CMC, Vellore | 1 | 12 | SQK-LSK110, EXP-NBD104 | FLO-MIN106 | 9h | 21.02.1 | 5.0.17 | High Accuracy | 0.852 | 2.56M |
| CMC, Vellore | 2 | 23 | SQK-LSK110, EXP-NBD104, EXP-NBD114 | FLO-MIN106 | 16h | 21.10.4 | 5.0.17 | High Accuracy | 1.52 | 4.92M |
| CMC, Vellore | 3 | 24 | SQK-LSK110, EXP-NBD104, EXP-NBD114 | FLO-MIN106 | 16h | 22.05.5 | 6.1.5 | High Accuracy | 0.594 | 858.92k |
| CMC, Vellore | 4 | 24 | SQK-LSK110, EXP-NBD104, EXP-NBD114 | FLO-MIN106 | 16h | 22.05.5 | 6.1.5 | High Accuracy | 0.556 | 3.29M |
| CMC, Vellore | 5 | 13 | SQK-LSK110, EXP-NBD104, EXP-NBD114 | FLO-MIN106 | 16h | 22.05.5 | 6.1.5 | High Accuracy | 0.508 | 5.31M |

Table B. *Salmonella* Typhi, non-Typhoidal *Salmonellae*, and non-*Salmonella* organisms included in the reference set for mapping the sequencing reads.

| **Organism** | **Accession/Source** | **Description** |
| --- | --- | --- |
| *S.* Typhi CT18 | NC_003198.1 | *Salmonella* Typhi chromosome |
| *S.* Typhi Ty2 | AE014613.1 | *Salmonella* Typhi chromosome |
| *S.* Typhi | 672572 (Pathogenwatch) | *Salmonella* Typhi chromosome |
| *S.* Typhi | 681355 (Pathogenwatch) | *Salmonella* Typhi chromosome |
| *S.* Typhi | Gurgaon01 (Pathogenwatch) | *Salmonella* Typhi chromosome |
| *S.* Typhi | Gurgaon02 (Pathogenwatch) | *Salmonella* Typhi chromosome |
| *S.* Typhi | SLT0626 (Pathogenwatch) | *Salmonella* Typhi chromosome |
| *S.* Typhi | SLT0892 (Pathogenwatch) | *Salmonella* Typhi chromosome |
| *S.* Typhi | SLT0291 (Pathogenwatch) | *Salmonella* Typhi chromosome |
| pHCM1 | NC_003384.1 | *Salmonella* Typhi plasmid |
| pHCM2 | NC_003385.1 | *Salmonella* Typhi plasmid |
| *S.* Paratyphi A | NZ_CP023508.1 | non-Typhoidal *Salmonella* (NTS) |
| *S.* Paratyphi B | CP000886.1 | non-Typhoidal *Salmonella* (NTS) |
| *S.* Paratyphi C | CP000857.1 | non-Typhoidal *Salmonella* (NTS) |
| *S.* Othmarschen | CP066260.1 | non-Typhoidal *Salmonella* (NTS) |
| *S.* Typhimurium | CP030029.1 | non-Typhoidal *Salmonella* (NTS) |
| *Pseudomonas lundensis* | CP075180.1 | Non-*Salmonella* species (NSS) |
| *Citrobacter freundii* | CP016762.1 | Non-*Salmonella* species (NSS) |
| *Enterobacter cloacae* | AP022274.1 | Non-*Salmonella* species (NSS) |
| *Cronobacter universalis* | CP012257.1 | Non-*Salmonella* species (NSS) |
| *Klebsiella pneumoniae* | CP052224.1 | Non-*Salmonella* species (NSS) |
| *Aeromonas veronii* | AP022264.1 | Non-*Salmonella* species (NSS) |
| *Escherichia coli* | CP099721.1 | Non-*Salmonella* species (NSS) |

Table C. *Salmonella* Typhi and non-Typhoidal *Salmonellae* included in the consensus trees. All isolate sequences downloaded from Pathogen Watch originated from a genomic surveillance study in India and were dated between 2018 and 2019.

| **Organism** | **Accession** | **Genotype (if *S.*Typhi)** |
| --- | --- | --- |
| *S*. Typhi Z10132 | ERR6118183 | 4.3.1.2 |
| *S*. Typhi G10113 | ERR5201201 | 4.3.1.2 |
| *S*. Typhi G10090 | ERR5201029 | 4.3.1.2 |
| *S*. Typhi B10179 | ERR5201077 | 4.3.1.2 |
| *S*. Typhi B10143 | ERR5201025 | 4.3.1.2 |
| *S*. Typhi Z10159 | ERR5200900 | 4.3.1.1 |
| *S*. Typhi T10255 | ERR5201322 | 4.3.1.1 |
| *S*. Typhi T10209 | ERR5201136 | 4.3.1.1 |
| *S*. Typhi R11685 | ERR5201054 | 4.3.1.1 |
| *S*. Typhi G10084 | ERR5200897 | 4.3.1.1 |
| *S*. Typhi S20472 | ERR5201157 | 4.3.1 |
| *S*. Typhi B10195 | ERR5201023 | 4.3.1 |
| *S*. Typhi Ty2 | AE014613.1 | 4.1 |
| *S*. Typhi CT18 | AL513382.1 | 3.2.1 |
| *S*. enterica subsp. enterica serovar Seftenberg | AP019692.1 |  |
| *S*. enterica subsp. enterica serovar Bareilly | CP034177.1 |  |
| *S*. enterica subsp. enterica serovar Weltevreden | CP040701.1 |  |
| *S*. enterica subsp. enterica serovar Infantis | CP052817.1 |  |
| *S*. enterica subsp. enterica serovar Typhimurium | NC_003197.2 |  |
| *S*. enterica subsp. enterica serovar Enteritidis | NZ_CP019681.1 |  |
| *S*. enterica subsp. enterica serovar Dublin | NZ_CP032387.1 |  |
