# Supplementary material for: Determining genotype and antimicrobial resistance of Salmonella Typhi in environmental samples by amplicon sequencing
Source: PLoS Negl Trop Dis. 2025 Jul 8;19(7):e0013211. doi: 10.1371/journal.pntd.0013211 (PMC12237024; doi:10.1371/journal.pntd.0013211)

Figure S1: Amplicon mapping for different primer pooling approaches. a) Single pool of all primers b) Primers split into two pools, Pool 1 and Pool 2 c) Primers split differently into two pools, Pool 3 and Pool 4

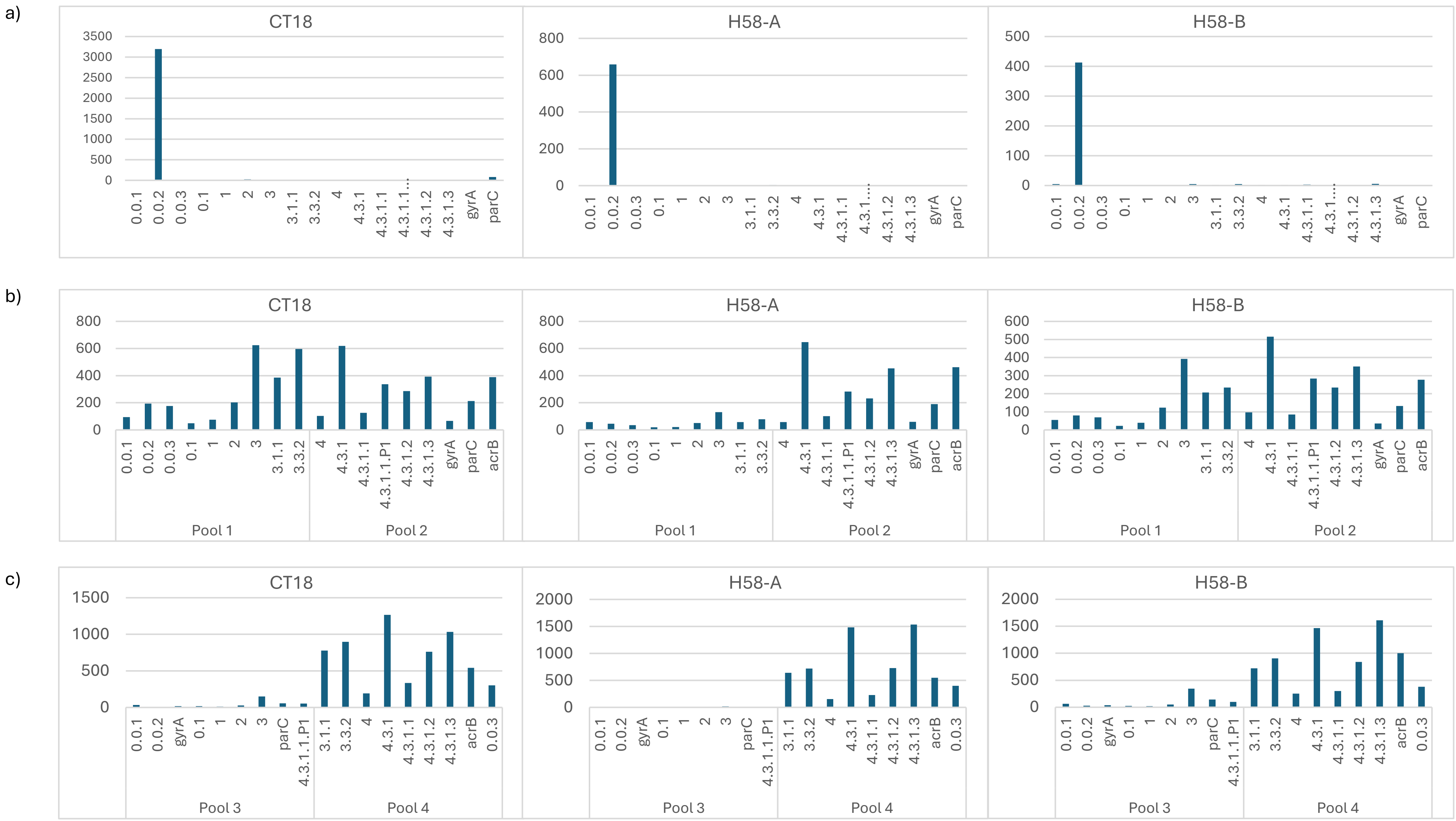

Supplement: S1 Fig — a) Single pool of all primers b) Primers split into two pools, Pool 1 and Pool 2 c) Primers split differently into two pools, Pool 3 and Pool 4. (PDF) [file pntd.0013211.s003.pdf]
